# Comparison of simulation software and contrastive training on ECG interpretation competence in newly graduated nurses

**DOI:** 10.3389/fmed.2025.1531308

**Published:** 2025-08-01

**Authors:** Zahra Kabiri, Reza Masoudi, Soleiman Kheiri, Shahram Etemadifar

**Affiliations:** ^1^Department of Nursing, Shahrekord University of Medical Sciences, Shahrekord, Iran; ^2^Department of Medical-Surgical, School of Nursing and Midwifery, Shahid Beheshti University of Medical Sciences, Tehran, Iran; ^3^Department of Biostatic, Shahrekord University of Medical Sciences, Shahrekord, Iran

**Keywords:** critical thinking, clinical competence, nurses, electrocardiogram, education

## Abstract

**Background:**

Improving the ability of nurses to interpret ECG and diagnose pathological disorders can be effective in preventing the complications of arrhythmias. Therefore, two of the most important indicators of nursing performance among newly graduated nurses for making clinical decisions are clinical competence and critical thinking. Therefore, this study was conducted to compare the effect of contrastive training and simulation software on the level of critical thinking and clinical competence in newly graduated nurses.

**Purpose:**

This quasi-experimental study was conducted in Shahrekord (Iran) to compare the effect of contrastive training and simulation software on the level of critical thinking and clinical competence in newly graduated nurses.

**Methods:**

A total of 66 newly graduated nurses participated in the study in two intervention and control groups. Inclusion criteria included providing informed consent to participate in the study, holding a bachelor’s degree in nursing, and having work experience of fewer than 24 months. The fundamentals of electrocardiogram were taught through one session, and then in the contrastive training group, in 5 sessions of 35 min, and in the software group. Data were collected before, immediately after, and 3 months after the intervention with the Clinical Competency and Critical Thinking Questionnaire in electrocardiogram interpretation.

**Results:**

The mean pre-intervention score of clinical competence was 6.36 ± 1.98 in the software group and 6.36 ± 2.50 in the contrastive training group, and immediately after the intervention 9.06 ± 1.48 in the software group and 9.74 ± 1.53 in the contrastive training group. Three months after the intervention, the mean score was 8.27 ± 1.89 in the software group and 8.87 ± 1.78 in the contrastive training group. There was a significant difference among all steps (*p* < 0.001). Before the intervention, the mean score of critical thinking was 5.61 ± 2.86 in the software group and 7.84 ± 3.15 in the contrastive training group, and immediately after the intervention 9.27 ± 1.93 and in the coping training 11.26 ± 2.90, and 3 months after the intervention 7.97 ± 2.17 and 10 ± 2.68 in the software group. The difference in scores on both variables at all intervals between the two groups was significant (*p* < 0.001). In both groups, there was a significant increase in both variables at all intervals, but the mean score was higher in the contrastive training group than in the simulation software group.

**Conclusion:**

Both contrastive training and simulation software promote critical thinking and clinical competence in interpreting electrocardiograms among newly graduated nurses, but the effects of training are mitigated over time and it is, therefore, necessary to take into consideration a longer training program using a simulation approach and contrastive training, especially in case of contrastive training.

## Background

Cardiovascular diseases are the leading cause of premature death across the world (1, 2) with an increasing rate (3), so their early diagnosis can help to better manage the symptoms of the diseases (4). In this regard, the most widely used method to examine heart conditions is electrocardiography (ECG) (5), which examines both muscle and electrical activity of the heart (2). ECG is used in some conditions, such as transient myocardial ischemia and cardiac arrhythmia, as a gold diagnostic standard, instead of many advanced diagnostic tests, and enables specialists to examine risks and symptoms (6, 7).

The ECG also enables decision-making in three stages of prehospital care, patient transfer to the appropriate hospital, and obtaining the information needed to provide PCI services (8). Therefore, due to the versatility, non-invasiveness, cheapness, and also ease of ECG in the rapid diagnosis of life-threatening arrhythmias, the acquisition of knowledge and skills regarding the test by nurses is essential (9).

Nurses have substantial responsibility in caring for patients undergoing cardiac monitoring (ECG). They are responsible for both the technical aspects of monitoring and clinical decision making based on monitoring information (10). Therefore, having sufficient knowledge and skills in this regard increases the quality of services. Today, the use of cardiac monitoring and 12 lead ECG, when combined with high nurse competence, makes it possible to diagnose not only coronary artery diseases but also arrhythmias (11, 12).

Besides that, one of the most important indicators of the clinical competence of nurses is their ability to accurately interpret the ECG, think critically and make critical decisions, and finally make clinical judgments based on it. ECG interpretation is an important area of clinical nursing education and regardless of the specialty of nurses, they are expected to have the competence to detect the rhythms in an ECG (13). However, research shows that nursing professionals often lack sufficient competence to detect the rhythms in ECGs (14). Breen et al. (15) reported that up to 33% of ECG interpretations contained some errors and up to 11% led to clinical mismanagement.

One of the important and influential components of nursing competence is the ability to think critically (16). According to great theorists such as Dewey, critical thinking is one of the most important educational principles of any country and any society needs people who enjoy high levels of critical thinking to achieve growth and prosperity (17, 18). The study of Tajvidi et al. (19) showed that the university curriculum is not effective for improving the critical thinking and clinical competence of nurses and it is necessary for clinical nursing planners to hold practical workshops in this field to develop critical thinking as an important and practical strategy for nurses to achieve optimal nursing care that is the ultimate goal of nursing.

Studies show that the application of the new methods leads to a better interpretation of the ECG (20). Although the results of the studies indicate that a particular educational method is not completely superior, but the use of an active method that is associated with more involvement of learners in educational programs and leads to better achievement of educational goals, is preferred to passive methods. Also, an advanced teaching method makes learning faster (21). It is therefore important to review and compare the effectiveness and efficiency of these methods for improving the skills of nurses, especially recently graduated ones.

One of these methods is the application of simulators. Simulation is a learning technique that, by creating all or part of a clinical experience in a safe environment, allows the individual to be educated through interactive activities without fear of personal weakness or patient harm. This method is effective and efficient for newly graduated nurses who have numerous worries and concerns because it mimics the facts in clinical settings and is designed in a way that creates processes for clinical decision-making and critical thinking by providing the conditions for roleplaying and utilizing tools such as interactive videos or mannequins (22).

Another effective method of learning is contrastive training, which leads to educational progress by changing the information structure. In this method, the learner acts as a diagnostician and instead of learning the concept of diseases, becomes acquainted with the characteristics that distinguish diseases from each other. This method of teaching is the exact search for similarities and differences between problems (23).

Studies show that newly graduated nurses do not have sufficient skills in interpreting ECGs. Reasons for this weakness include the complexity of ECGs’ interpretation and volatile content, as well as insufficient curricular teaching. However, nurses are the first people who can play a key role in diagnosing heart arrhythmias in patients with heart problems. Early diagnosis of heart problems has a direct impact on the quality of life of these patients (11, 12).

A report by Jeffries et al. (24) compared the effectiveness of two methods of teaching nurses how to perform a 12-lead ECG. The traditional method included a self-study module and a brief lecture by an instructor. The second method used the same content with an interactive, multimedia CD-ROM supplemented with a self-study module. Overall results indicated that both groups were satisfied with their learning experience and had a similar improvement in their ability to interpret ECGs (24). A study of the effectiveness of a Web-based ECG teaching method vs. a traditional lecture method using a pretest/post-test experimental design with undergraduate nursing students suggested that knowledge about ECGs among students in the web based teaching group was significantly lower than that of students in the traditional lecture group (t = −3.527, *p* < 0.01). Conversely, the ability to interpret ECG recordings was significantly higher among students in the web-based teaching group (t = 2.839, *p* < 0.05) (25).

Information is limited about Iranian newly graduated nurses’ knowledge of ECGs. Therefore, there is a critical need for clinical instructors to train newly graduated nurses in the appropriate hospital units on ECG interpretation and to evaluate the effects of this training to ensure actual improvement.

It should be considered that each teaching method has its advantages and disadvantages. There is no threat to patient safety in the use of simulation software, the clinical situations and training schedule can be controlled by the instructor, and there is the possibility of feedback and correction of activity during the performance while using the software. However, developing such software can be costly. In contrastive training, people can solve the problem by carefully examining the similarities and differences. Considering the contradictory evidence from previous research on the effectiveness of different educational techniques, the objective of this study was to conduct a rigorous comparison of two contrasting approaches, namely, simulation software and contrastive training, to ascertain and analyze their differential impacts on educational achievement. The primary goal of this study was to compare the efficacy of simulation software and contrastive training in enhancing ECG interpretation skills for newly graduated nurses; additionally, it sought to preliminarily explore the underlying factors that might explain any disparities in the effectiveness of these two distinct teaching approaches. Recognizing the distinctions between these methods could significantly impact future research endeavors, leading to improvements in educational strategies and ultimately enhancing the clinical competency of newly graduated nurses, thereby ensuring the application of more effective methodologies in their practice.

## Materials and methods

A quasi-experimental, interventional study involving newly graduated nurses from Hajar and Kashani Hospitals in Shahrekord, Iran, was conducted over a period spanning from 2021 to 2022.

This study was conducted and the findings were reported according to the STROBE checklist (26). From July to August 2021, the intervention was implemented in both groups involved in the study. The ethics code IR.SKUMS.REC.1400.102 was issued for the study protocol by the Vice-Chancellor for Research and Technology of Shahrekord University of Medical Sciences, and the objectives of the study were explained to the participants, and then informed consent to participate in the study was obtained from them. The confidentiality of participants’ identities was also strictly observed.

In this study, the study population consisted of newly graduated nurses in Hajar and Kashani Hospitals of Shahrekord. Participants included 66 eligible newly graduated nurses working in various shifts who were selected through purposive sampling. Inclusion criteria included providing informed consent to participate in the study, holding a bachelor’s degree in nursing, and having work experience of fewer than 24 months (27). Participants who voluntarily withdrew from the study or withdrew from the paramedic program were excluded.

**Figure 1:**
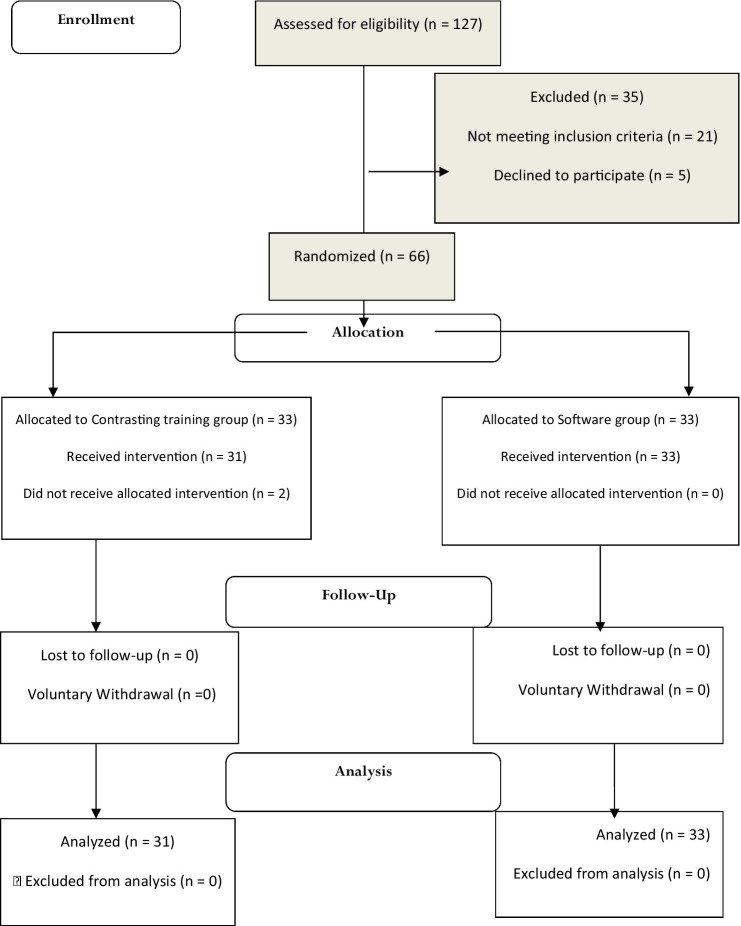
The CONSORT flow diagram of the study.

Therefore, given the 95% confidence interval (CI) and 90% test power, the number of samples was calculated at 30 according to the sample size calculation formula. To counteract the possible dropouts, 10% of the calculated sample size (3 individuals for each group) was added to the sample size and finally, the study was conducted with the participation of 66 individuals assigned to two groups by random allocation (Figure 1).

Data collection tools consisted of two questionnaires on clinical competence and critical thinking in interpreting electrocardiograms. The clinical competence questionnaire used in this study was developed by Coll-Badell et al. (28). The questionnaire consists of 2 parts; the first part consists of demographic information and the second part contains 12 items on ECG. The questionnaire’s ICC was calculated at 0.869 and its CVI at 0.87 (28). A 15-four-choice item, a questionnaire designed by Alamrani et al. (29) was administered to assess participants’ ability of critical thinking on ECG interpretation. The content index of the items was rated from 0.75 to 1 and the average credit index of the items was obtained to 0.95. Given the use of this questionnaire for the first time in Iran in this study, the questionnaire was first translated into Persian by an English language expert and then translated back into English and approved by another person who was an English language expert. To confirm the reliability of the questionnaire, a test–retest was performed so that the questionnaire was administered to 10 newly graduated nurses, and a week later it was completed again by the same nurses and its Cronbach’s alpha was calculated at 82.6.

### Intervention

The training was led by a researcher with guidance and oversight from a nursing faculty member, to ensure the validity and quality of the training. During one session, all participants were given general information about the ECG and the general characteristics of a normal ECG. Participants were then randomly divided into two groups of 33 individuals each using a random allocation rule. The questionnaires were administered to both groups before the intervention. Then, the nurses in the contrastive training group were divided into three groups of 11 individuals, and the coping training program was presented in five 35-min sessions over 2 weeks (30).

During the training sessions, participants in the group could freely discuss and exchange ideas, and the researcher guided the session using an interactive approach. The questionnaires were completed by the participants immediately after the sessions. The questionnaires were administered to participants again 3 months after the intervention. During the follow-up period, the researcher assigned 1 day of the week to answer the problems and questions of the participants in person. This issue was also followed up by phone 1 day of the week. In the contrastive training group, slides including three electros with the same diagnosis and another electro with a different diagnosis (as deviant) were presented to participants. Participants were asked to compare and contrast electros with each other. For example, a slide containing four ECGs, three left ventricular hypertrophies, and one normal electro, was shown to them and then they were asked to describe the similarities and differences. The second slide contained four electros, two electros showed ventricular hypertrophy, and two electros ischemic heart disease (a confusing disorder not previously seen), and participants were asked to compare the characteristics of each and distinguish them.

Participants were encouraged to find similarities and differences between categories, and where participants made mistakes and were unable to articulate differences, the researcher reminded them of differences. This process was repeated until all diagnostic categories were completed. An application that could be installed on cell phones with operating systems of Android or Apple’s iOS was procured for the simulation software group. The process of purchasing and licensing the software went through legal steps. The content of the software was approved by the faculty members of the School of Nursing. Its software-related capabilities were also confirmed by a medical informatics expert, then it was approved by the IT Department of Shahrekord University of Medical Sciences. The software was also provided to the other group of participants.

The software included the topic of ECG, the properties of ECG paper, the shapes and naming of the waves, sinus rhythms and sinus arrhythmias, atrial rhythms, junctional rhythms, ventricular rhythms, atrioventricular node blocks, and heart blocks. The application was provided to the nurses and in a virtual session in which all the participants of this group attended, according to the schedule presented in the group, the participants were asked to study a specific topic through the software, and on day 3, 5, 7, 9, 11 after receiving the program, questions appropriate to the material studied were raised and discussed in the group. If necessary, the researcher provided guidance and advice.

Questionnaires were completed by the participants immediately after the sessions and 3 months (20). During the follow-up period, the researcher determined 1 day of the week to answer the participants’ questions in person. Also, on 1 day of the week, the participants in the software group were contacted by phone to remind them of studying the content of the software. Due to the nature of this study, the nurses could not be blinded. To ensure the accuracy of our primary outcome assessment, which was ECG interpretation competence, evaluators were blinded to patient’s group assignment.

### Data analysis

SPSS (version 21) was used to conduct data analysis. Inter-group and intra-group comparisons were performed by independent t-test, chi-square test, and repeated measures analysis of variance.

The significance level (P) in all analyses was considered to be <0.05.

## Results

A total of 66 newly graduated nurses participated in this study and were assigned to two groups: contrastive training (*n* = 33) and software training (*n* = 33). Two people from the coping training group were excluded from the study due a lack of volunteering to cooperate with the study because of shift schedule interference, and finally, the data of 31 people in the contrastive training group and 33 ones in the simulation software group were analyzed (Table 1).

**Table 1:** Frequency of some demographic characteristics in the two intervention and control groups.

Variable		Group				Significance level
		Simulation software group; *N* (%)		Contrastive training group; *N* (%)		
Gender	Male	9	27.3	4	12.9	0.15
	Female	24	72.7	27	87.1	
	Total	33	100	31	100	
Working section	Orthopedics	2	6.1	2	6.5	0.97*
	Surgery	4	12.1	1	3.2	
	Urology	4	12.1	4	12.9	
	Corona ward	5	15.2	5	16.1	
	CCU	4	12.1	4	12.9	
	ICU	9	27.3	8	25.8	
	Emergency	3	9.1	4	12.9	
	ENT	1	3	1	3	
	Neurology	1	3	2	6.5	
	Total	33	100	31	100	

^*^Fisher's exact test. The research findings regarding the demographic characteristics of the study samples showed that out of 64 participants in the study, in terms of gender, in the software training group, 9 out of 33 nurses were male (27.3%) and 24 were female (72.7%). In the confrontational training group, out of 31 participating nurses, 4 were male (12.9%) and 27 were female (87.1%). The table shows that the majority of nurses in the confrontational training group (87.1) and the software group (72.7%) are women. Also, based on Fisher's exact test, there is no significant difference between the confrontational training group and the software group in terms of gender (*p* = 0.15). The results of the study on the work area of nurses showed that the highest number of participating nurses in the ICU department, 27.3% in the software group and 25.8% in the confrontational training group, and the lowest number in the ENT department in the confrontational training group with 3%, and in the software group, the neurology and ENT departments with 3% each. The results showed that there was no significant difference in terms of the nurses' work area between the two groups (*p* = 0.97). All nurses participating in the study had a bachelor's degree. Also, none of the nurses participating in the study had a previous training history in electrocardiogram interpretation.

The results of the research regarding the departments of nurses showed that the highest number of nurses worked in the ICU (27.3% in the simulation software group and 25.8% in the contrastive training group) and the lowest number in the ENT department in the contrastive training group (3%) and neurology and ENT departments in the simulation software group (3% in each department). The results showed that there was no significant difference in this regard between the two groups (*p* = 0.97). All nurses had a bachelor’s degree. Also, none of the nurses had any previous training in ECG interpretation (Table 2).

**Table 2:** The mean age and work experience of participants in contrastive training group and simulation software group.

Group	Simulation software	Contrastive training	Significance level
Age	24.52 ± 1.03	24.52 ± 1.06	0.99
Work experience	10.46 ± 4.82	10.21 ± 4.75	0.86

### Critical thinking

The repeated measures ANOVA results showed that there was a significant increase in the score of critical thinking in both groups (*p* < 0.001). The mean score decreased at 3 months postintervention compared to the mean score attained immediately after the intervention, but was significantly higher than the pre-intervention mean score.

The interaction effect of group and time showed that there was no difference in score changes during the study between the two groups. In other words, in both groups, the score of critical thinking post-intervention increased compared to pre-intervention (*p* < 0.001) but showed a significant decrease 3 months post-intervention (*p* < 0.001) (Table 3).

**Table 3:** The mean scores of critical thinking during the study in contrastive training group and simulation software group.

Stage		Group		Significance level
		Simulation software	Contrastive training	
Pre-intervention		5.61 ± 2.86	7.84 ± 3.15	0.004
Post-intervention		9.27 ± 1.93	11.26 ± 2.9	0.001
3 months post-intervention		7.97 ± 2.17	10 ± 2.68	0.001
Intragroup *p* value	Pre-intervention × post-intervention	<0.001	<0.001	^*^<0.001 ^**^0.79
	Pre-intervention × 3 months post-intervention	<0.001	<0.001	
	Post-intervention × 3 months post-intervention	<0.001	<0.001	

^**^Interaction effect of group × time. ^*^Effect of time.

### Clinical competence

The repeated measures ANOVA results showed that there was a significant increase in clinical competence scores during the study in both groups (*p* < 0.001). The interaction effect of time and group showed that there was no difference in score changes between the two groups during the study.

In other words, in both groups, the post-intervention clinical competence score showed a significant increase compared to the pre-intervention clinical competence score (*p* < 0.001), but showed a significant decrease at 3 months post-intervention (*p* < 0.001) (Table 4).

**Table 4:** The mean scores of clinical competence in electrocardiogram interpretation during the study in group and simulation software group.

Stage		Group		Significance level
		Simulation software	Contrastive training	
Pre-intervention		6.36 ± 1.98	6.36 ± 2.50	0.78
Post-intervention		9.06 ± 1.48	9.74 ± 1.53	0.06
3 months post-intervention		8.27 ± 1.89	8.87 ± 1.78	0.21
Intragroup *p* value	Pre-intervention × post-intervention	<0.001	<0.001	^*^<0.001 ^**^0.79
	Pre-intervention × 3 months post-intervention	<0.001	<0.001	
	Post-intervention × 3 months post-intervention	<0.001	<0.001	

^**^Interaction effect of group × time. ^*^Effect of time.

## Discussion

The results of this study showed that the age, gender, work experience, and department of nurses in both groups and during all steps of the study, including pre-intervention and post-intervention, were not associated with their levels of critical thinking and clinical competence in ECG interpretation.

Our results regarding clinical competence in ECG interpretation showed that the mean preintervention score of clinical competence in the simulation software group and the contrastive training group was 6.36 with no statistically significant difference between the two groups (*p* = 0.78); according to the criteria of Coll-Badell et al. (28), people who attain a score of less than 7.5 have not reached the minimum level of clinical competence in ECG interpretation, so our results indicate that the newly graduated nurses in this study did not have the minimum level of clinical competence in ECG interpretation before the intervention.

Our results also showed that the mean score of clinical competence increased significantly immediately after the intervention in both groups (*p* = 0.01). The results showed that no significant difference was observed in increased clinical competence scores immediately after the intervention between the two groups (*p* = 0.06), but the mean scores were higher in the contrastive training group than in the simulation software group. In the simulation software group, the nurses stated that group discussions and activities in the form of a virtual group, following the study of educational software topics, provided the possibility of learning from mistakes and led to better teaching of clinical issues. Arrogante et al. (31) reported that clinical simulation made educational errors related to clinical techniques or methods, basic knowledge, and standards in clinical practice possible. In the study of Rahimpour et al. (6), which examined the clinical competence of emergency and emergency services nurses, the level of competence in both groups was lower than the predetermined minimum. In the study of Coll-Badell et al. (28), in which the competence in interpreting ECGs among nurses in Spain was studied, the competence of participants score was calculated at 8.6 ± 1.1, which, albeit higher than the minimum score, had a significant relationship with education history. In our study, the mean score increased after training in both methods.

In the study of Mobrad (14) in Saudi Arabia on ECG interpretation competence among emergency service students conducted using online questionnaires with 137 first to fourth-year students, two cardiology courses were offered in the second semester and the fourth semester of internship in the studied university; 64% of the participants obtained a score of over than 7.5, which was significantly related to the cardiology training course and the student’s grade point average (*p* < 0.001) (14). That study also found that an increased mean score was directly correlated to passing the training course. One of the reasons for the lack of nurses’ adequate knowledge of interpreting ECGs is that although nurses consider ECG interpretation as one of their duties, in general, they believe that doctors are the main interpreters of ECG, therefore, less involvement of nurses in ECG interpretation and infrequency of ECG interpretation among nurses may lead to their insufficient professionalism in interpreting ECGs (15).

A cross-sectional study of the reversible causes of cardiac arrest with emphasis on nursing qualifications with clinical simulation in undergraduate nursing students in Spain with 106 third year students (2020), showed that more than 90% of nursing students achieved a high level of satisfaction and 85.6% of nursing students acquired the necessary nursing competence for satisfactory management of the causes of cardiac reversibility (31). In that study, the simulation method also increased the mean score of students. The simulation method in that study consisted of four scenarios derived from four critical conditions that were performed by four groups of nursing students. Although this method of simulation improved the participants’ clinical and communication skills and competencies, the participants expressed a high level of anxiety and stress during performing the scenarios. In the study of Lak et al. (32) on the usage of simulation software and lectures to increase the knowledge of ECG interpretation among nurses, the results showed a significant difference in the mean (±standard deviation) score after the intervention in the simulation software group. In that study, the simulation software also improved ECG interpretation (32).

In that study, simulation software could be installed on a computer, which was installed on the ward computer and made available to ward nurses at any time. Although the software was consistently available, it might be less frequently used due to the heavy workload of nurses on each shift. In the study of Alamrani et al. (29) in Egypt to compare the effect of simulation-based and traditional teaching methods on critical thinking abilities and self-confidence in electrocardiogram interpretation in 30 nursing students, the results showed that after training, a significant increase in scores was observed, although no significant difference was observed in the scores between the two groups.

The simulation method in that study included a computer mannequin that simulated human functions and included a cardiac monitor, which showed hemodynamics, heart rhythms, and vital signs. In this study, information was evaluated only immediately after training. Other limitations of that study included the small number of participants in each group and the fact that all participants were classmates, which may affect the results. In the present study, a larger number of participants were included and the nurses were not selected from one single setting.

In the review article of Adib-Hajbaghery and Sharifi (33) to investigate the effect of simulation training on the development of critical thinking of nurses and nursing students in Kashan, eight studies reported that simulation training positively affects critical thinking skills. However, eight studies reported that simulation had no effect on critical thinking. The studies reviewed in that article reported that the results on the effects of simulation on the critical thinking in nurses and nursing students are contradictory.

The results of the present study showed that simulation software training promotes critical thinking among nurses, and although its rate decreased over time, it still showed a significant increase compared to the average pre-intervention score on critical thinking.

Taken together, different methods of educational programs, regardless of their type, if implemented effectively and efficiently for participants, can lead to the promotion of nursing critical thinking ability (34, 35). However, studies have shown that critical thinking is influenced by age, year of studies, and educators’ use of problem-solving methods (29, 36).

This research provides valuable results which can be effectively integrated into continuing education initiatives. The process of continuing education leverages the latest research findings to shape curriculum development, enhance teaching methodologies, and evaluate the efficacy of educational interventions; these improvements in education, in turn, serve as a foundation for and directly inform advancements in healthcare policy, nursing practices, and ultimately, the quality of patient care.

The results indicated a reduction in the nurses’ competence following a three-month intervention, highlighting the need for future studies to confirm the integration of these findings into practical nursing; future studies would benefit from testing these methods on more experienced nurses, exploring hybrid models, or even using AI-powered ECG training tools to enhance the validity and generalizability of the research conclusions.

The results of this study showed that the age, gender, work experience, and department of nurses in both groups and during all steps of the study, including pre-intervention and post-intervention, were not associated with their levels of critical thinking and clinical competence in ECG interpretation. This finding is consistent with previous research indicating that individual demographic factors alone are not the primary determinants of ECG interpretation competence (6, 28).

Our results also showed that the mean score of clinical competence increased significantly immediately after the intervention in both groups (*p* = 0.01). The results showed that no significant difference was observed in increased clinical competence scores immediately after the intervention between the two groups (*p* = 0.06), but the mean scores were higher in the contrastive training group than in the simulation software group. These findings are in line with previous studies that have demonstrated the effectiveness of structured educational interventions, such as simulation and contrastive training, in improving ECG interpretation skills (14, 31).

Importantly, the discussion of continuing education and in-service training is supported by several studies emphasizing its role in maintaining and enhancing clinical competence and critical thinking among nurses (37, 38). Continuing education not only ensures the updating of professional knowledge and skills, but also contributes to the sustained improvement of clinical performance over time (38).

One of the key findings in our study was the decrease in clinical competence and critical thinking scores 3 months after the intervention. This result is consistent with previous research indicating that the effects of short-term educational interventions may diminish over time, highlighting the need for periodic refresher courses or booster interventions (38, 39). Based on these findings, we suggest that a three-month interval could serve as a practical benchmark for planning future refresher training and ongoing professional development activities for nurses (39).

Moreover, a review of the literature shows that evaluating the impact of educational interventions at multiple time points (immediately after training and at follow-up) provides a more accurate picture of the durability of learning (29, 38). Therefore, future studies should consider assessing outcomes at various intervals to better understand the long-term effects of educational programs.

In summary, our findings indicate that both educational approaches, when properly designed and implemented, can lead to significant improvements in clinical competence and critical thinking among nurses. However, to sustain these gains, it is essential to design evidence-based continuing education programs with regular intervals, as supported by the literature (37, 39).

### Study limitations

In our study, only newly graduated nurses were included and therefore the assessment of work experience’s effect on the interpretation of ECG in different departments could not be carried out. As well, the grade point average of the graduates and the universities where the participants studied were not taken into consideration.

It is therefore recommended that other training methods be compared with contrastive training and simulation software training to achieve definitive results so that a safe and effective method can be delineated to create memorization and strengthen critical thinking and clinical competence for nurses through in-service training in ECG interpretation. Also, all male and female nurses at any age working in different departments with any work experience are recommended not to consider them needless of in-service education; in this regard, nursing managers need to include all nurses in their policies and planning.

## Conclusion

The results showed the desirable effect of simulation software and contrastive training in strengthening the critical thinking and clinical competence of newly graduated nurses in ECG interpretation in the short term, but the implementation of this method could not play a role in the retention and stability of critical thinking and clinical competence in ECG interpretation of nurses and develop nurses’ knowledge of ECG interpretation in the long run. It is suggested that future research should explore the perspectives of various stakeholders regarding the effectiveness of ECG interpretation curricula, and further comparative studies should be conducted focusing on the ECG interpretation training methods employed by successful nursing schools to better understand best practices for educating nursing students.

## Data Availability

The original contributions presented in the study are included in the article/supplementary material, further inquiries can be directed to the corresponding author.
